# Enhanced Solar Cell Conversion Efficiency Using Birefringent Liquid Crystal Polymer Homeotropic Films from Reactive Mesogens

**DOI:** 10.3390/ijms141121319

**Published:** 2013-10-25

**Authors:** Gwomei Wu, Li-Hang Hsieh, How-Wen Chien

**Affiliations:** Institute of Electro-Optical Engineering, Chang Gung University, Kweisan, Taoyuan 333, Taiwan; E-Mails: leon.hsieh.18@facebook.com (L.-H.H.); chienhowwen@gmail.com (H.-W.C.)

**Keywords:** liquid crystal polymer, energy conversion efficiency, reflectivity

## Abstract

Novel birefringent liquid crystal polymer homeotropic films have been coated on semiconductor solar cells to improve the effective incident sunlight angles. The liquid crystal polymer precursor, based on reactive mesogens, is fluidic and flows like liquid. It would distribute uniformly on the solar cell sample surface by any traditional coating technique. The birefringence for light, due to the liquid crystal retardation properties, manipulated the optical length and the deflection of incident light, thus allowed an increase in the energy conversion efficiency. The expensive sunlight tracking systems could be avoided. The processing parameters can be tuned such as different mesogen concentrations and plate speeds of spin-coating. The results showed that the solar cell conversion efficiency was improved from 14.56% to 14.85% at an incident sunlight angle of 15°. It was further improved from 13.40% to 13.81% when the angle was 30°. The interesting angular dependency on solar cell efficiency enhancement has been evaluated.

## 1. Introduction

Photovoltaic solar cells with high energy conversion efficiency have attracted much attention in the last decade, mainly due to the increasingly higher cost in natural energy resources and stronger environmental protection concern [1–5]. In addition to the advancements in the semiconductor technology, novel optical design to minimize reflection, thus maximize the incident sunlight absorption, becomes essential for the success of this widely growing industry [6]. It is noted that the prevailing technology has been surface texturing by wet chemical etching, which utilizes strong acidic and/or alkaline solutions to engrave the surface of silicon. For example, the different etching rates for the (100) and (111) oriented planes by potassium hydroxide allow anisotropic pyramids to be developed on single crystalline Si(100). The etched pyramids can redirect some of the reflected light for further absorption, and thus improve the sunlight conversion efficiency [7,8].

For lower cost polycrystalline silicon, the different sized crystalline grains are randomly oriented. The reactive ion etch and isotropic etch by specialized formulation are still being investigated for the best results. Basu *et al.* reported the use of sodium hydroxide and sodium hypochlorite solution at an elevated temperature for large area solar cells [9]. Tao *et al.* proposed a monolayer of microscale silica particles partially immersed in the spin-on-glass film [10]. The surface texturing effect was thus achieved by coating, and no physical damage to the silicon was performed. On the other hand, with the advancement in reactive mesogen chemistry, anisotropic liquid crystal polymer film can be designed for optical birefringence [11,12]. Liquid crystal polymer can provide homeotropic alignment characteristics [13]. The control of light passage becomes readily possible. It is therefore interesting to develop a one-step coating technology that exhibits antireflection to implement the surface texturing effect without actually using the strong chemical etching methods [14,15].

In this paper, birefringent liquid crystal polymer homeotropic films have been coated on semiconductor solar cells, for the first time, to improve the effective angles of the incident sunlight. The optical retardation properties of the cured reactive mesogens could be carefully controlled by the appropriate processing parameters. The liquid crystal polymer precursor was fluidic and was distributed uniformly by the traditional spin-coating technique. The novel process was quite simple and has become fairly reliable. The processing parameters included different mesogen concentrations, plate speeds of spin-coating, and ultraviolet (UV) curing conditions. The coated semiconductor solar cells were investigated by the solar simulator model YSS-50A and a quantum efficiency measurement system. The current–voltage (I–V) and power–voltage (P–V) curves were measured at the different sunlight incident angles for the solar cell efficiency enhancement evaluation.

## 2. Results and Discussion

**Figure 1:**
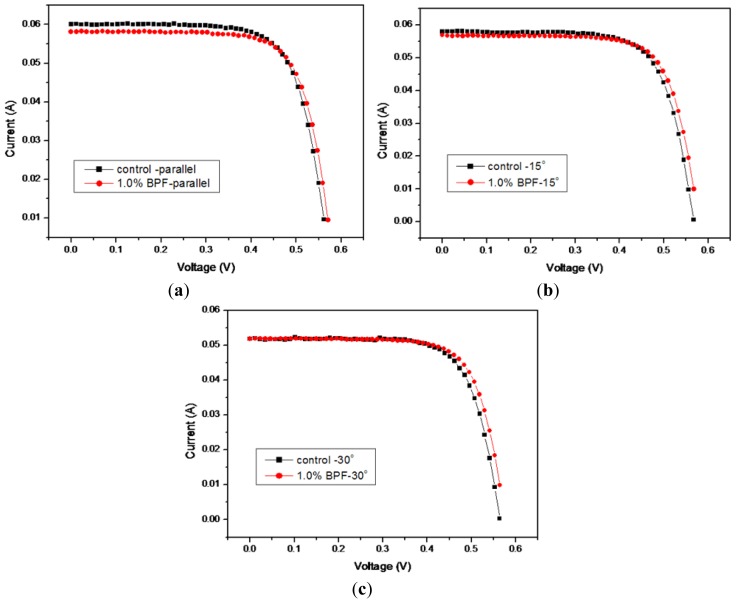
The measured I–V curves for the coated solar cell samples using 1% reactive mesogen liquid crystal precursor solution. The light incident angle was 0° for (**a**); 15° for (**b**); and 30° for (**c**). The control solar cell sample data are also included for the ones without the liquid crystal polymer coatings.

**Figure 2:**
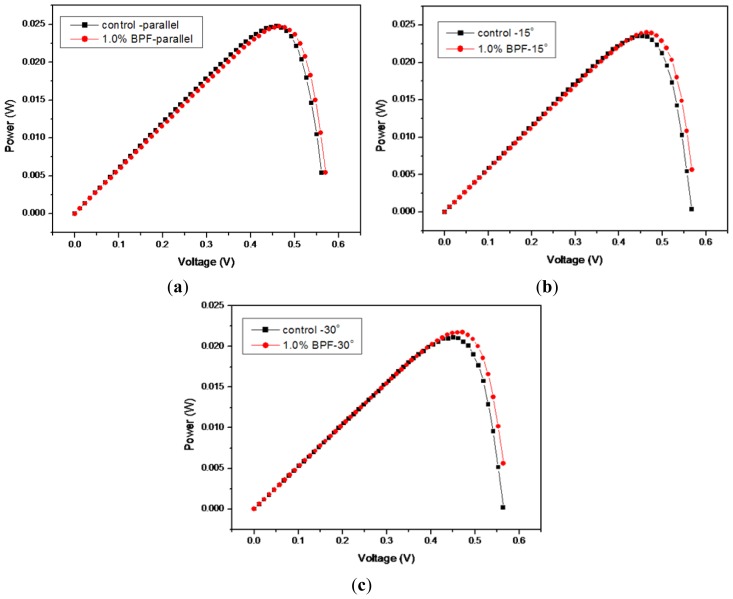
The measured P–V curves for the coated solar cell samples using 1% reactive mesogen precursor solution. The light incident angle has been 0° for (**a**); 15° for (**b**); and 30° for (**c**). The control solar cell sample data are also included for the ones without the liquid crystal polymer coatings.

The birefringent liquid crystal polymer homeotropic thin films have been coated on silicon solar cells to improve the effective angles of the incident sunlight. Figure 1 shows the measured current–voltage (I–V) curves for the coated solar cell samples using 1% reactive mesogen precursor solution. The control solar cell sample data are also included for the ones without any liquid crystal polymer coatings. In addition, Figure 2 shows the measured power–voltage (P–V) curves for the coated solar cell samples, also using 1% reactive mesogen precursor solution. The effects of the different operating voltages on cell currents are displayed along with the cell power values. Both the coated and the control (non-coated) samples were measured in the three different incident angles for solar cell performance evaluation. The short-circuit current (*I*_sc_) was slightly reduced and the open-circuit voltage (*V*_oc_) was slightly increased.

The energy conversion efficiency of the 1%-coated silicon solar cell is approximately equal to that of the control cell when the incident angle is 0° or in parallel with the normal direction of the cell surface. However, it exhibited a higher value at 14.85% for the 1%-coated cell when the incident angle was increased to 15°. This represented an enhancement of 1.97% as compared with the control cell at 14.56%. When the cells were tested at an even higher incident angle of 30°, the degree of enhancement was even more obvious at 3.05%, showing an improvement from 13.40% to 13.81%. In addition, it was verified that the silicon solar cell experimental samples showed very stable results after 1 month at room temperature. However, long-term stability and deteriorative evaluation using a temperature and relative humidity control chamber is needed for practical future implentation. Some organic-inorganic hybrid system can be also designed for this type of application.

**Figure 3:**
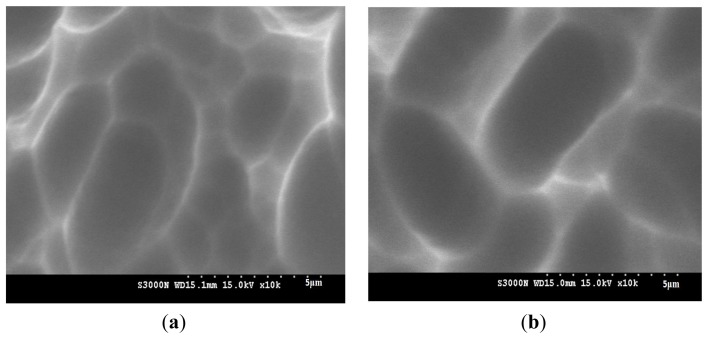
SEM surface micrographs of the silicon solar cell samples: (**a**) the control sample without polymer coating; and (**b**) the polymer-coated sample using 1% reactive mesogen precursor solution.

The thickness of the polymer film was estimated at 50–100 nm, depending on the liquid crystal polymer precursor concentration and the curing condition. It has been noted that the solar cell conversion efficiency would be degraded with an increase in sunlight incident angle. That is why a conventional sunlight tracking system can be employed to improve the conversion efficiency close to that of the direct sunlight. But the system would impose high hardware assembly cost, and part of the precious electrical power can be depleted during the operation. It has been also suggested to coat nano-particles on the surface of a stationary solar cell, and the effective light incident angle could be improved [16]. However, it is still difficult to distribute the nano-particles on the solar cell surface uniformly. The curing solution may become uneven between two nano-particles by any coating process. In this study, the optical retardation properties of the cured reactive mesogens could be carefully controlled to provide high anisotropy in optical transmittance. The easy-flowing characteristics of the precursor solution made it possible to distribute itself uniformly on the solar cell surface by a traditional spin-coating technique. Figure 3 displays the SEM surface micrographs of the silicon solar cell samples. In the control sample, the round-shape crater-like microstructures on the silicon surface were likely caused by acidic isotropic etching from removing saw damages. It was largely attributed to the combined effect of chemical reaction and transport limit by diffusion. The mechanism of acidic saw-damage etching has been fully discussed in the literature [17]. On the other hand, the polymer-coated sample revealed uniform coverage of the etched silicon surface. No discernible aggregates or pits could be observed. The anisotropic birefringence manipulated the optical length and the deflection of incident light, which attracted more light into the active semiconductor substrate and thereby improved the energy conversion efficiency, especially at the higher incident angles. The degradation in solar cell conversion efficiency along an increased sunlight incident angle was thus decelerated.

The interesting angular dependency results on solar cell conversion efficiency are summarized in Table 1 for all the coated cell samples, with the different precursor concentrations ranging from 0.1%–5.0%. Although the liquid crystal polymer film was highly transparent, it could still absorb a fairly small amount of incident light, costing the efficiency data by 0.02%–0.11%. On the other hand, a very low liquid crystal polymer precursor concentration (<0.1%) solution had almost no effect on the solar cell performance.

A much higher precursor concentration (>10%) made the uniform distribution of the polymer homeotropic film more difficult due to a higher viscosity during the curing process. The diffusion of the residual solvent at the polymer–silicon interface becomes more complicated when the surface polymer has been firstly fully cured by UV light exposure. It has been suggested that some adjustments on the curing conditions are needed, including pre-baking temperature and time, and UV light power density and its time distribution. The haze appearance also indicated possible voids in the thicker film structure. The optimal precursor concentration was thus suggested at 1%–2% for our system. The conversion efficiency enhancement data, calculated from the coated cells and the control cells, are listed in Table 2. The slightly negative values were caused by the absorption from the liquid crystal polymer films. The actual energy conversion efficiency data were thus slightly inhibited by this absorption.

**Figure 4:**
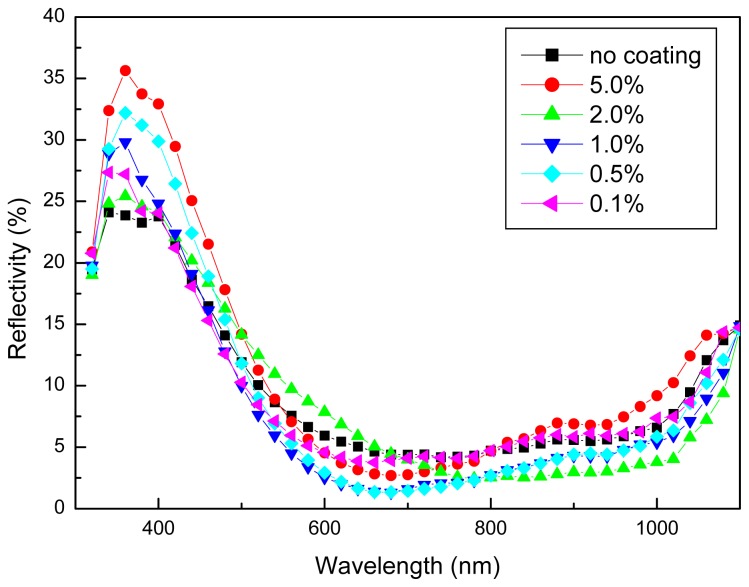
The reflectivity spectra for all the coated and non-coated solar cell samples in the wavelength range of 300–1100 nm.

So far, we have demonstrated that the novel birefringent liquid crystal polymer coatings showed good enhancement in energy conversion efficiency at inclined incident angles for photovoltaic silicon solar cells. A higher degree of improvement was observed with a larger incident angle. It will be interesting to evaluate the findings with the cells’ antireflective characteristics. Figure 4 shows the reflectivity spectra for all the coated and non-coated (control) solar cell samples in the wavelength range of 300–1100 nm. It has been demonstrated that the 5%-precursor cell sample exhibited the highest reflectivity, especially in the short wavelength range of 300–500 nm. This is in accordance with the lower energy conversion efficiency results in Tables 1 and 2. The lower reflectivity for the 1%-precursor cell sample near 400–800 nm was thus reported for the more efficient silicon solar cells. The improved manipulation and redirection of the incident light, thus lower reflectivity, attracted more sunlight photons into the semiconductor substrates and therefore enhanced the energy conversion efficiency. A more versatile solar cell system could be implemented by the proper design of the birefringent liquid crystal polymer thin film structures, especially for the larger incident angle applications.

## 3. Experimental Section

The silicon solar cells were firstly cleaned and prepared in the size of 15 × 15 mm^2^. A liquid crystal polymer precursor solution of the reactive mesogens with the vertical alignment characteristics (Merck RMS3015, Darmstadt, Germany) of the specified concentration was coated on the cell samples using a spin-coater, with a two-stage spinning process, 500 rpm (round per minute) for 10 s and 3000 rpm for 30 s. The precursor solution is pre-filtered and the UV-cured films have been designed to exhibit optical retardation properties with a +*C* optical symmetry axis [18,19]. Its room temperature viscosity is about 2–3 cPs, and can vary with the resin content in the solution. Several transflective liquid crystal display devices have been proposed from this liquid crystal polymer in the literatures [19,20]. A soft bake was carried out at 100 °C for 80 s to dry out the organic solvent of propylene glycol monomethyl ether acetate (PGMEA). The coated samples were then cured by an ultraviolet (UV) aligner (UniVex-500, Univex Company, Jhonghe, Taiwan) for polymerization under a nitrogen purge. The UV wavelength was 365 nm, and the exposure time was about 3 min. The power density has been estimated to be about 8 mW/cm^2^. Five different precursor concentration levels were studied at 5%, 2%, 1%, 0.5% and 0.1%.

After UV-curing, the ordinary refractive index (*n*_o_) of the liquid crystal polymer film is about 1.53 (λ = 550 nm) and the extraordinary refractive index (*n*_e_) of the film is 1.67. The liquid crystalline phase transition clearing point was estimated at 96 °C. The anisotropic optical birefringence (Δ*n*) has been about 0.14. It was also noted that the surface free energy was around 45–47 mN·m^−2^.

The effects of the birefringent liquid crystal polymer films on the energy conversion efficiency of the silicon solar cells were investigated by the solar simulator model YSS-50A (Yamashita Denso Corporation, Tokyo, Japan) and a quantum efficiency measurement system (SCS100, Zolix, Beijing, China). The current–voltage (I–V) and power–voltage (P–V) curves were measured at three different sunlight incident angles at 0°, 15° and 30° at room temperature. The incident angle was defined as 0° when the incident light was perpendicular to the surface of the solar cell samples. In addition, the multifunctional solar cell scan system SCS100 (Zolix, Beijing, China) was also used to reveal the reflectivity spectra under the wavelength range of 300–1100 nm. The derived sunlight energy conversion efficiency data were then evaluated according to the various processing and measurement parameters.

## 4. Conclusions

In this paper, we have designed and fabricated novel birefringent liquid crystal polymer-coated silicon solar cells. The prepared liquid crystal polymer precursor solution was fluidic and could flow like liquid. It would be distributed uniformly on a solar cell surface by the traditional spin-coating technique. It has been successfully demonstrated that the 1%-precursor liquid crystal polymer-coated solar cell samples exhibited an enhancement of 1.97% at the incident angle of 15°. The enhancement was further increased to 3.05% at a larger incident angle of 30°. The improvements have been in accordance with the lowered reflectivity spectra results. Thereby, the costly chemical etching and/or texturing could be replaced or eliminated in this growing industry. The expensive sunlight tracking system might not be needed any more. The objective in achieving higher solar cell energy conversion efficiency with a more environmentally friendly technology could thus be implemented by a proper design of the birefringent liquid crystal polymer film coating structures. Further investigations will be needed to evaluate deteriorative and long-term stability characteristics

## Figures and Tables

**Table 1:** The incident angle dependency results on solar cell conversion efficiency for all the coated and control (non-coated) cell samples. The precursor concentration ranged from 0.1% to 5.0%.

Incident angle (°)	Conversion efficiency (%)					

	Precursor 5.0%	Concentration 2.0%	Concentration 1.0%	Concentration 0.5%	Concentration 0.1%	Control
0	14.74	14.80	14.83	14.75	14.82	14.85
15	14.81	14.77	14.85	14.62	14.48	14.56
30	13.79	13.56	13.81	13.53	13.47	13.40

**Table 2:** The efficiency improvement data were calculated from the coated cells and the control cells. The slightly negative values were caused by the absorption from the organic polymer films. The intrinsic energy conversion efficiency data were thus slightly inhibited by this absorption.

Incident angle (°)	Improvement (%)				

	Precursor 5.0%	Concentration 2.0%	Concentration 1.0%	Concentration 0.5%	Concentration 0.1%
0	−0.73	−0.33	−0.14	−0.69	−0.17
15	1.73	1.46	1.97	0.43	−0.52
30	2.94	1.21	3.05	0.96	0.50
